# Transcriptomic and Metabolomic Analysis Following *LmRab11A* Knockdown Reveals Its Role in Metabolic Regulation and Molting in *Locusta migratoria*

**DOI:** 10.3390/insects17080803

**Published:** 2026-08-03

**Authors:** Mureed Abbas, Yiyan Zhao, Abdul Basit, Jianqin Zhang, Xuemei Qin, Yunhe Fan

**Affiliations:** 1 Modern Research Center for Traditional Chinese Medicine, The Key Laboratory of Chemical Biology and Molecular Engineering of Ministry of Education, Shanxi University, Taiyuan 030006, China; 2 Shanxi Bethune Hospital, Shanxi Academy of Medical Sciences, Third Hospital of Shanxi Medical University, Tongji Shanxi Hospital, Taiyuan 030032, China; 3 State Key Laboratory of Ecological Pest Control for Fujian and Taiwan Crops, Key Laboratory of Biopesticides and Chemical Biology, MOE College of Plant Protection, Fujian Agriculture and Forestry University, Fuzhou 350002, China; 4 School of Life Science, Shanxi University, Taiyuan 030006, China

**Keywords:** *Rab11A*, RNAi, transcriptomics, metabolomics, *Locusta migratoria*

## Abstract

Rab proteins are key members of the Ras superfamily that regulate vesicular trafficking in eukaryotic cells, ensuring accurate cargo transport and cellular homeostasis. To investigate the functional role of Rab proteins, RNA interference (RNAi) was employed to silence *LmRab11A* in *Locusta migratoria*, followed by integrated transcriptomic and metabolomic analyses. Transcriptomic profiling identified 54 downregulated and 71 upregulated genes upon *LmRab11A* knockdown. Based on Log2 fold change values and statistical significance, 15 downregulated genes were selected for further analysis. RT-qPCR validation confirmed that 9 of these genes were significantly downregulated following *LmRab11A* silencing. Subsequent RNAi-mediated knockdown of these 9 genes revealed that silencing of only *LOCMI11062* (*β-tubulin*) resulted in 100% mortality and severe morphological defects in locust nymphs, indicating its critical role in development. Metabolomic analysis identified 11 significantly altered metabolites, including 8 exhibiting increased abundance and 3 exhibiting decreased abundance. Spearman correlation analysis between the 9 downregulated genes and the altered metabolites suggested potential associations between 6 metabolites and specific genes. Collectively, these findings demonstrate that suppression of *LmRab11A* disrupts downstream gene expression and alters the metabolite network, thereby impairing growth and development.

## Simple Summary

In our previous study, we found that *LmRab11A* plays a critical role in the molting process of the migratory locust. To further investigate the function of *LmRab11A*, in this study, we employed RNA interference (RNAi) to silence the *LmRab11A* gene, followed by integrated transcriptomic and metabolomic analyses. Transcriptomic analysis combined with RT-qPCR revealed that 9 genes were significantly downregulated following *LmRab11A* knockdown. Subsequent RNAi-mediated knockdown of these 9 genes showed that silencing only *LOCMI11062* (*β-tubulin*) led to arrested molting and 100% mortality in locust nymphs, a phenotype reminiscent of that caused by *LmRab11A* knockdown, indicating the essential role of *β-tubulin* in development. Metabolomic analysis identified 11 significantly altered metabolites, 8 exhibiting decreased abundance and 3 exhibiting increased abundance. Spearman correlation analysis between the 9 downregulated genes and the metabolite suggested potential associations between 6 metabolites and specific genes. Taken together, these findings indicate that *LmRab11A* functions as an important regulatory factor influencing gene expression, metabolic homeostasis, and developmental processes in *Locusta migratoria*, thereby contributing to normal growth and molting. This study expands our understanding of the molecular mechanisms underlying *LmRab11A* function and identifies potential targets for the development of novel locust management strategies.

## 1. Introduction

Ras protein superfamily consists of small protein GTPases that regulate essential cellular processes [1]. Rab proteins that belong to the Ras superfamily are important regulators of cellular transport in eukaryotes. These proteins regulate vesicle traffic between different cellular compartments, and ensure the precise delivery of cargo such as lipids, proteins, and other essential molecules [2]. Rab proteins are fundamental in modulating cellular homeostasis via vesicular trafficking and help in regulating a wide range of physiological processes, such as signal transduction, nutrient uptake, and waste removal [3,4]. Rab protein functions by altering between an active GTP-bound state (often found on membranes) and an inactive GDP-bound state (usually found in the cytosol). This process is regulated by guanine nucleotide exchange factors (GEFs) that promote the exchange of GDP for GTP and GTPase-activating proteins (GAPs) that catalyze the hydrolysis of GTP to GDP [5]. Rab proteins in their active state interact with different effector molecules, such as tethering factors and motor proteins, and facilitate vesicle transport along cytoskeletal elements and ensure precise delivery of cargo [4,6]. Rab11 regulates the recycling of endosomes and the delivery of proteins from endosomes to the cell membrane [7]. It plays critical roles in key cellular processes such as cytokinesis, epithelial cell polarization, and ciliary membrane formation [8,9]. Given the diverse and crucial functions of Rab proteins, metabolomic and transcriptomic studies are required to further elucidate their functional dynamics and pathways in cellular physiology.

The migratory locust, *Locusta migratoria* (*L. migratoria*), is a major agricultural pest worldwide and also serves as an important model organism for studying insect physiology. Due to its complex physiological traits, *L. migratoria* is used as an excellent model for investigating the functional roles of Rab proteins [10,11]. Despite being one of the most destructive pests globally, the molecular mechanisms underlying the biology of *L. migratoria* are largely underexplored. The integration of transcriptomic and metabolomic analyses could offer a strong approach to elucidate the molecular basis of physiological traits in locusts.

Knockdown of Rab proteins, such as LmRab11A in *L. migratoria*, provides an opportunity to investigate the underlying molecular mechanisms that regulate their physiological traits. Transcriptomic and metabolomic analyses following *LmRab11A* knockdown revealed its influence on gene expression and metabolic profiles, indicating its roles in regulating expression of key genes and metabolic pathways. This approach also provided insights into how vesicular transport processes contribute to the adaptive and survival strategies of locusts [11,12]. The functional roles of Rab proteins in locusts may have broader implications for the investigation of vesicular transport in other eukaryotes, given the evolutionary conservation of Rab proteins among eukaryotes [11].

In our previous study, silencing of *LmRab11A* in *L. migratoria* resulted in significant mortality [11]; however, the underlying mechanisms remained unresolved. Building on these findings, the present study aims to systematically investigate the effects of Lm*Rab11A* knockdown on gene expression and metabolomic profiles in *L. migratoria*. By identifying differentially expressed genes and altered metabolites associated with Lm*Rab11A* silencing, we seek to elucidate the molecular pathways through which this Rab protein regulates critical cellular processes essential for locust physiology. These results will not only advance our understanding of *L. migratoria* biology but will also provide broader insights into conserved cellular mechanisms across diverse biological systems.

## 2. Materials and Methods

### 2.1. Insect Rearing

The eggs of *L. migratoria* were acquired from a locust breeding facility in Cangzhou, Hebei Province, China, and maintained at 30 ± 2 °C, relative humidity of 40 ± 5% and photoperiod of 14:10 (light:dark) hours in the insectariums at the Institute of Applied Biology, Shanxi University, Taiyuan, Shanxi, China. The nymphs were placed in well-ventilated rearing cages (25 cm × 25 cm × 25 cm) at population densities of 50 to 500 insects/cage and fed with fresh wheat seedlings. Bran was added once the locust grew to third instar. To maintain developmental synchronization, individuals were transferred to different cages immediately after each molt.

### 2.2. Synthesis of dsRNA

Double-stranded RNAs (dsRNA) targeting *LmRab11A* gene and green fluorescent protein (*EGFP*) as a control were designed and synthesized. Primers for dsRNA synthesis were designed with T7 promoter sequence (TAATACGACTCACTATAGGG) at 5′ end online using the E-RNAi web service (https://dsrna-engineer.cn/, accessed on 4 September 2022) [11]. The template for dsRNA synthesis was amplified by PCR using cDNA from fifth-instar nymphs using 2× Taq Master Mix (TaKaRa, Tokyo, Japan). The PCR products were then purified using a E.Z.N.A^TM^ Gel Extraction Kit (Omega Bio-Tek, Inc., Norcross, GA, USA) and used as a template for synthesis of dsRNA using T7 RiboMAX^TM^ Express RNAi System (Promega, USA). The concentration of each purified dsRNA was determined using a Nanodrop 2000 spectrophotometer (Thermo Fisher Scientific, Waltham, MA, USA). The integrity of purified dsRNA was assessed by 1% agarose gel electrophoresis, and dsRNA was stored in elution buffer at −20 °C until further use.

### 2.3. RNA Interference (RNAi) of LmRab11A Gene

RNAi injections were performed using a dose of 10 μg per nymph. Injections were carried out by injecting into the hemocoel of 2-day-old fifth-instar (N5D2) nymphs through the second abdominal segment. In parallel, locusts injected with equal doses of *EGFP* dsRNA (ds*EGFP*) were used as controls using microinjectors (Gaoge, Shaoxing, China). 15 insects were injected; after 24 h of injection, integuments were dissected and homogenized in TRIzol for extraction of total RNA. First-strand cDNA was synthesized from 1 μg of each total RNA sample using M-MLV reverse transcriptase (TaKaRa, Japan). RT-qPCR was performed to investigate the relative expression of *LmRab11A* after RNAi. The expression level of the target gene was normalized to the steady-state level of elongation factor 1-alpha (*EF1a*). 5 biological replicates from each control and treatment were analyzed.

### 2.4. Total RNA Isolation and Transcriptome Sequencing

Total RNA was isolated from 2-day-old fifth-instar (*N5D2*) *L. migratoria* nymphs (average body weight was 0.47 ± 0.06 g) at 24 h after injection with 10 μg of target dsRNA using RNAiso Plus Reagent (TaKaRa, Tokyo, Japan) according to the manufacturer’s protocol. RNA quality and integrity were evaluated by 1% agarose gel electrophoresis, while RNA concentration and purity were determined using a Nanodrop 2000 spectrophotometer (Thermo Fisher Scientific, Waltham, MA, USA).

mRNA was purified from total RNA using poly-T oligo-attached magnetic beads. First-strand cDNA was synthesized, and second-strand cDNA synthesis was subsequently performed. Remaining overhangs were converted into blunt ends via exonuclease/polymerase activities. After adenylation of 3′ ends of DNA fragments, NEBNext Adaptor with hairpin loop structure was ligated to prepare for hybridization. The library fragments were purified with AMPure XP system (Beckman Coulter, Beverly, MA, USA). Then, 3 μL USER Enzyme (NEB, Ipswich, MA, USA) was used with size-selected, adaptor-ligated cDNA at 37 °C for 15 min followed by 5 min at 95 °C before PCR. Then, PCR was performed with Phusion High-Fidelity DNA polymerase, Universal PCR primers and Index (X) Primer. At last, PCR products were purified (AMPure XP system), and library quality was assessed on the Agilent 2100 Expert software (v B.02.08). The libraries were sequenced on an Illumina NovaSeq platform (Illumina, Inc., San Diego, CA, USA) to generate 150 bp paired-end reads, according to the manufacturer’s instructions.

### 2.5. Functional Annotation

The raw reads were further processed with a fastp software (v 0.22.0). The adapter sequences and low-quality sequence reads were removed from the data sets. Raw sequences were transformed into clean reads after data processing. These clean reads were then mapped to the reference genome sequence. Only reads with a perfect match or one mismatch were further analyzed and annotated based on the reference genome. HISAT2 software (v 2.2.1) was used to map with reference genome. Gene function was annotated based on the following databases: Nr (NCBI non-redundant protein sequences) (https://www.ncbi.nlm.nih.gov/protein/, accessed on 4 September 2022); Pfam (Protein family) (http://pfam.xfam.org/, accessed on 4 September 2022); KOG/COG (Clusters of Orthologous Groups of Proteins) (http://www.ncbi.nlm.nih.gov/KOG/; https://www.ncbi.nlm.nih.gov/COG, accessed on 4 September 2022); Swiss-Prot (a manually annotated and reviewed protein sequence database) (http://www.uniprot.org/, accessed on 4 September 2022); KO (KEGG Ortholog database) (http://www.genome.jp/kegg/, accessed on 4 September 2022); and GO (Gene Ontology) (http://www.geneontology.org/, accessed on 4 September 2022).

### 2.6. Differential Expression Analysis

Gene expression levels were estimated as fragments per kilobase of transcript per million mapped reads (FPKM). Differential gene expression analysis between the dsRNA-treated and control groups was performed using DESeq2 (v1.42.0). DESeq2 provides statistical routines for determining differential expressions in digital gene expression data using a model based on negative binomial distribution. The resulting *p*-values were adjusted using Benjamini and Hochberg’s approach for controlling the false discovery rate (FDR). Genes with an adjusted *p*-value < 0.01 and fold change ≥ 2 found by DESeq2 were assigned as differentially expressed.

### 2.7. dsRNA Knockdown Confirmation

Based on the transcriptomic analysis, a total of 54 genes were identified as significantly downregulated following *LmRab11A* RNAi, using the criteria of fold change ≥ 2 and *p*-value < 0.01. To prioritize genes for RT-qPCR validation, these 54 genes were ranked in descending order according to their absolute Log_2_-transformed fold-change values (|Log_2_FC|), and the top 15 genes with the largest fold-changes were selected. For each candidate gene, specific RT-qPCR primers and dsRNA synthesis primers were designed using Primer Premier 5 software and the E-RNAi web service (https://dsrna-engineer.cn/, accessed on 4 September 2022). Then, 10 μg dsRNA targeting each gene was injected into 2-day-old fifth-instar (N5D2) nymphs of *L. migratoria*. For each treatment, 15 insects were injected using a micro-injector (Gaoge, Shaoxing, China). At 24 h post-injection, the integument of the second abdominal segment was dissected from each insect. The dissected tissues from every 5 insects were pooled as 1 biological replicate, yielding 3 independent biological replicates per treatment (*n* = 3, with 5 insects per replicate). Each pooled sample was homogenized in RNAiso Plus (TaKaRa, Japan) for total RNA extraction. First-strand cDNA was synthesized from 1 μg of total RNA per replicate using M-MLV reverse transcriptase (TaKaRa, Japan). RT-qPCR was performed to investigate the relative expression of each gene. The target gene expression level was normalized to the expression of the internal reference gene *EF1α* (elongation factors 1alpha) which exhibited the most stable expression at different stages and in different tissues [13,14,15]. Melting curve analysis was performed to confirm the specificity of amplification. The relative gene expression level was calculated by the $2^{-\Delta c_{T}}$ method.

### 2.8. Phenotypic Analysis and Mortality Bioassay

To evaluate the biological functions, RNAi of 9 down-regulated DEGs was performed. dsRNA for each of the 9 DEGs (with a dose of 6 μg per nymph) was injected into the hemocoel of 2-day-old third-instar (N3D2) nymphs using a micro-injector (Gaoge, Shaoxing, China) through the second abdominal segment. In parallel, *L. migratoria* injected with equal doses of ds*EGFP* was used as the control. For each treatment, 30 insects were injected, and mortality along with developmental defects was monitored at 24 h time intervals for a period of 15 days. Further RNAi was conducted using the previously described methodology [11].

### 2.9. Sample Preparation for Metabolomics

To determine the metabolic changes in *L. migratoria*, a dose of 10 μg ds*LmRab11A* was injected into the hemocoel of 2-day-old fifth instar (N5D2) nymphs using micro-injector (Gaoge, Shaoxing, China), administered through the second abdominal segment. In parallel, *L. migratoria* injected with equal doses of EGFP dsRNA (ds*EGFP*) were used as the control. After 24 h post injection, locust nymphs were dissected to collect integument tissues. 8 biological replicates were prepared, and 10 individuals’ integuments were pooled for each biological replicate.

### 2.10. Detection and Analysis of Metabolites by Untargeted Metabolomics

For metabolite extraction, integument tissues pooled from 10 *L. migratoria* nymphs were immediately frozen in liquid nitrogen and ground into a fine powder (*n* = 8). Approximately 50 mg of tissue was extracted with 200 μL of methanol by vortexing for 30 s, followed by centrifugation at 13,000× *g* for 20 min at 4 °C. The supernatant was transferred to a fresh microcentrifuge tube and centrifuged again at 13,000× *g* for 10 min at 4 °C. The clarified supernatant evaporated to dryness under a gentle stream of nitrogen gas, and the residue was reconstituted in 100 μL of methanol, after a final centrifugation at 13,000× *g* for 20 min at 4 °C. Finally, the supernatant was transferred to an autosampler vial insert for UPLC-MS/MS analysis. Additionally, a 10 µL aliquot of each sample was pooled to prepare a quality control (QC) sample. Chromatographic separation was performed on an Acquity UPLC HSS T3 column (1.8 µm, 2.1 mm × 100 mm) at a column temperature of 35 °C. The mobile phase consisted of 0.1% formic acid in water (solvent A) and 0.1% formic acid in acetonitrile (solvent B), with a flow rate of 0.2 mL/min. The injection volume was 5 µL.

The raw MS data were processed using MS-DIAL software (v 4.70) for peak detection, alignment, and filtering. To evaluate the overall metabolic differences and identify differentially accumulated metabolites, supervised orthogonal partial least-squares discriminant analysis (OPLS-DA) was performed. The model performance was assessed using a seven-fold cross-validation to calculate R2 and Q2 values, where R2 reflects the explanatory power of the model and Q2 indicates its predictive capability. To further validate the model robustness and prevent overfitting, a permutation test with 200 random permutations was conducted, yielding the intercept values of R2 and Q2. A lower Q2 intercept value suggests better model reliability and a reduced risk of overfitting. Additionally, the variable importance in projection (VIP) scores was derived from the OPLS-DA model to estimate each metabolite’s contribution to the observed group discrimination. Metabolites fulfilling the criteria of VIP > 1 and *p* < 0.05 (based on Student’s *t*-test) were defined as significantly altered metabolites. Subsequently, the differential metabolites were identified using the MetDNA algorithm (http://metdna.zhulab.cn, accessed on 22 September 2022), which employs a metabolic reaction network-based recursive annotation strategy to achieve large-scale and reliable metabolite identification. Annotation was further validated by matching the MS/MS spectra against public databases, including HMDB, KEGG, and an in-house standard library.

### 2.11. Statistical Analysis

Statistical comparisons between two groups were performed using the two-tailed Mann–Whitney U test. In the figures, asterisks indicate significant differences (* *p* < 0.05; ** *p* < 0.01; *** *p* < 0.001).

## 3. Results

### 3.1. Transcriptomics Analysis of Differentially Expressed Genes in L. migratoria

**Figure 1:**
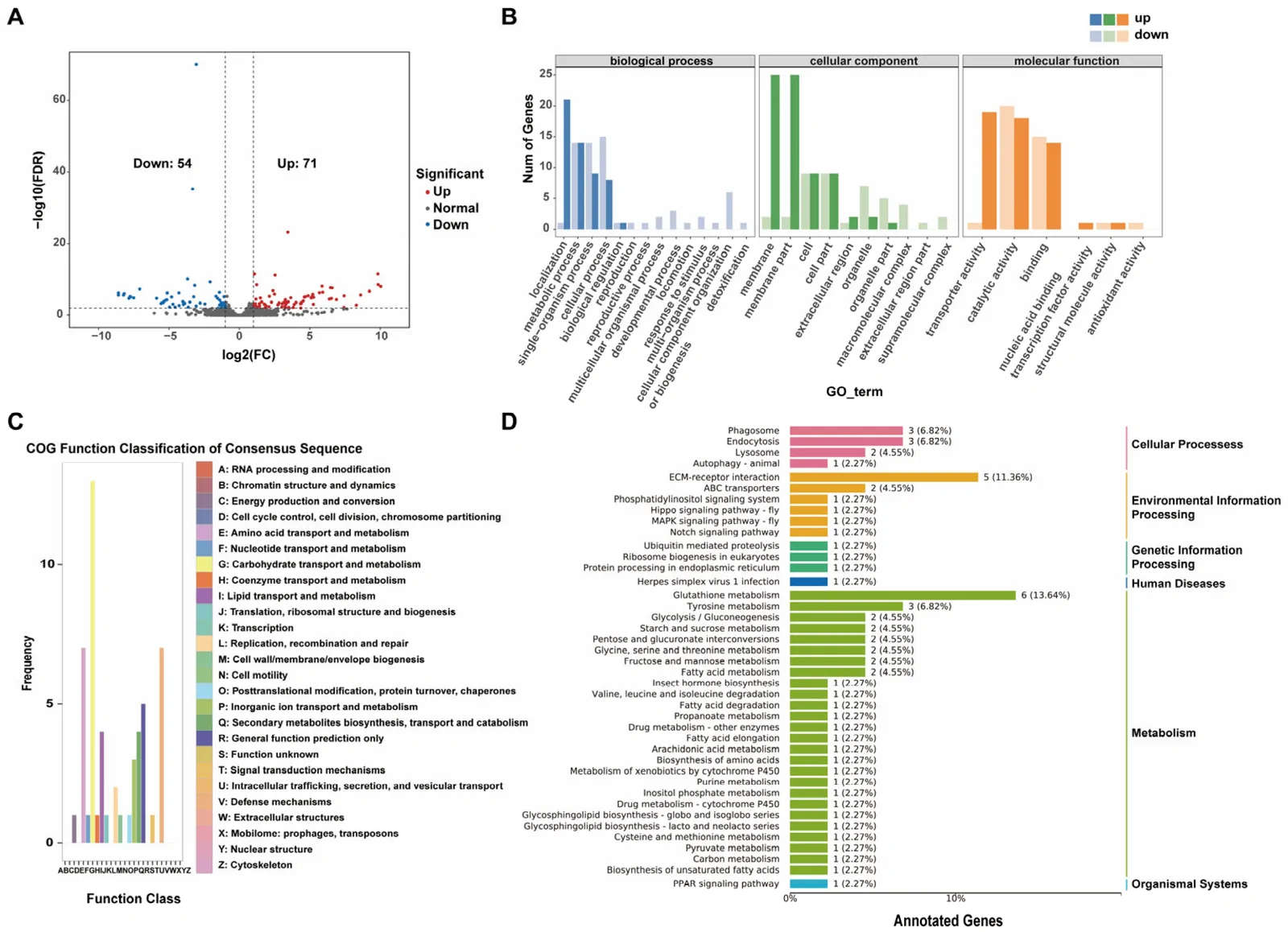
Analysis of differentially expressed *LmRab11A* genes based on transcriptomics. (**A**) Volcano plot of differentially expressed genes in the integument. (**B**) Gene ontology (GO) assignment. (**C**) Clusters of orthologous groups (COG) classification and (**D**) KEGG classification of annotated genes.

The clean reads from each sample were aligned to the designated reference genome, with alignment rates ranging from 75.66% to 82.64%. Based on the alignment results, we performed analyses of alternative splicing prediction, gene structure optimization, and novel gene discovery. A total of 7904 novel genes were identified, of which 2732 were successfully annotated with functional information. Comparative transcriptomic analysis between the ds*LmRab11A*-treated and control groups identified 125 differentially expressed genes (DEGs), including 71 upregulated and 54 downregulated genes (Figure 1A).

To further analyze the function, all the DEGs were allocated to the following three specific gene ontology (GO) categories: biological process, cellular component and molecular function. Results showed 30 subcategories within these categories as follows: 14 subcategories for biological process, 10 for cellular component and 6 for molecular function. Within these subcategories, ‘Localization’ was the most abundant sub-category in the biological process category followed by ‘metabolic process’; ‘membrane’ and ‘membrane part’ were the most abundant in the cellular component category, while ‘transport activity’ dominated the molecular function category (Figure 1B).

Furthermore, to predict the putative Rab protein functions, a COG analysis was performed. The results showed that carbohydrate transport and metabolism was the largest sub-category of cellular component followed by amino acid transport and metabolism and defense mechanism (Figure 1C). Pathway enrichment analysis showed that the DEGs were the most enriched in the ‘Glutathione metabolism’ pathway, followed by the ‘ECM receptor interaction’ pathway. In addition, these DEGs were divided into the following 6 KEGG categories: cellular process, environmental information processing, genetic information processing, human disease, metabolism and organismal system. Among these, metabolism and environmental information processing contained the most pathways. These 6 categories were further subdivided into 41 subcategories. Statistical analysis of these 41 subcategories revealed that the largest number of DEGs were involved in glutathion metabolism (13.64%), followed by ECM–receptor interaction (11.36%), phagosome (6.82%), and endocytosis (6.82%) (Figure 1D). The identified DEGs and enriched pathways highlight critical biological processes and potential targets for further research on locust physiology and development.

### 3.2. Identification and Expression Analysis of Differentially Expressed Genes in L. migratoria

**Figure 2:**
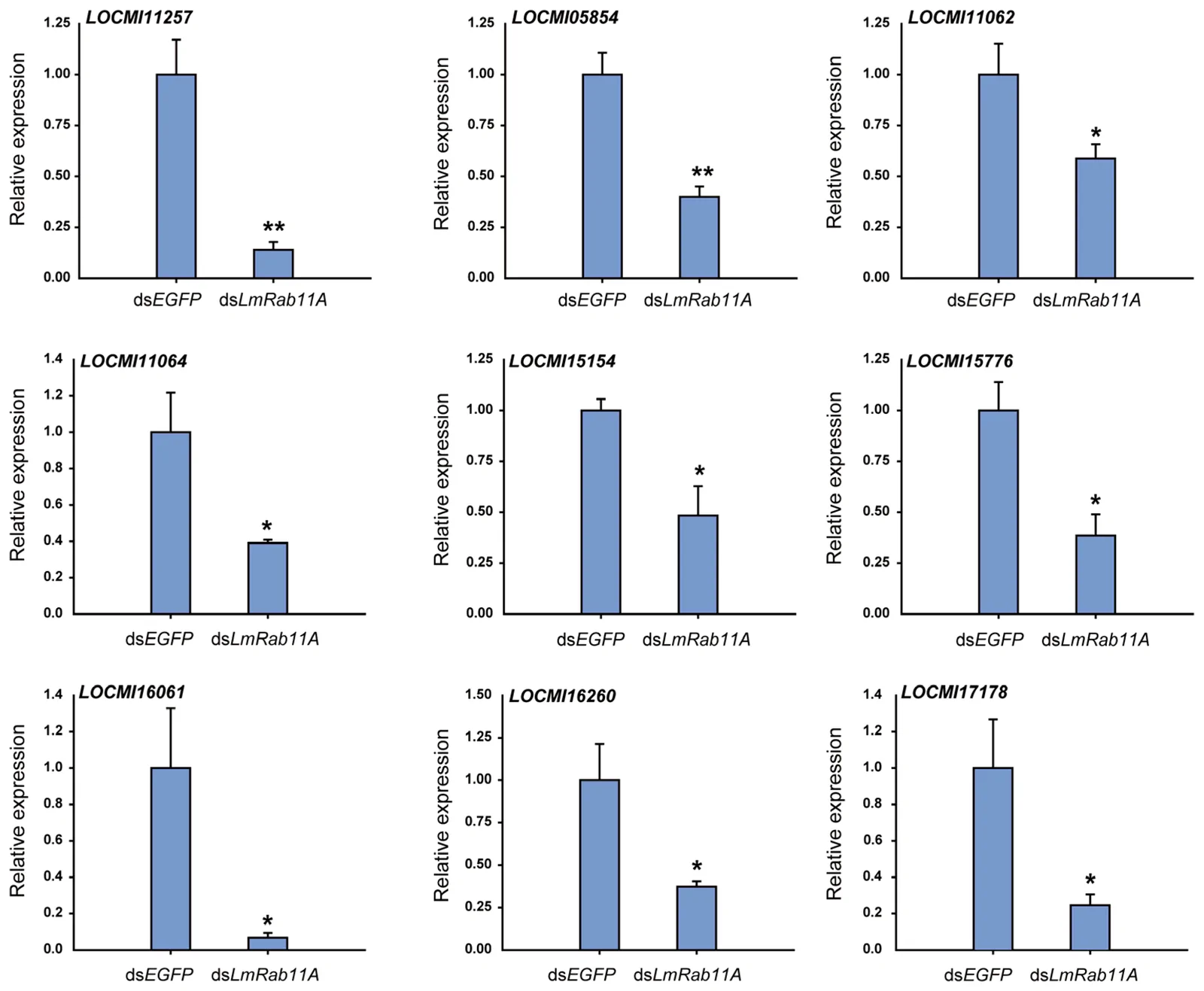
Relative expression of 9 genes after ds*LmRab11A* was injected. Statistical analysis was performed using Mann–Whitney U test. Asterisks represent significant difference (* *p* < 0.05; ** *p* < 0.01).

Of the 54 down-regulated genes, the 15 most significantly down-regulated ones were selected for validation by RT-qPCR. The results revealed that 9 out of these 15 genes were significantly down-regulated in the integument tissues (Figure 2) (gene-specific primer sequences are provided in Table S1, while the genes exhibiting no significant down regulation are presented in Figure S1). Among them, one energy production and conversion-related gene (*LOCMI16061*), one lipid transport and metabolism-related gene (*LOCMI15776*), two post-translational modification, protein turnover, chaperones-related genes (*LOCMI15154* and *LOCMI16260*), one signal transduction mechanisms-related gene (*LOCMI17178*), one intracellular trafficking, secretion, and vesicular transport-related gene (*LOCMI11257*), and three Cytoskeleton-related genes (*LOCMI11062*, *LOCMI11064*, and *LOCMI5854*) were significantly down-regulated in the integuments.

### 3.3. Phenotypic Analysis After RNAi of 9Down-Regulation Genes

**Figure 3:**
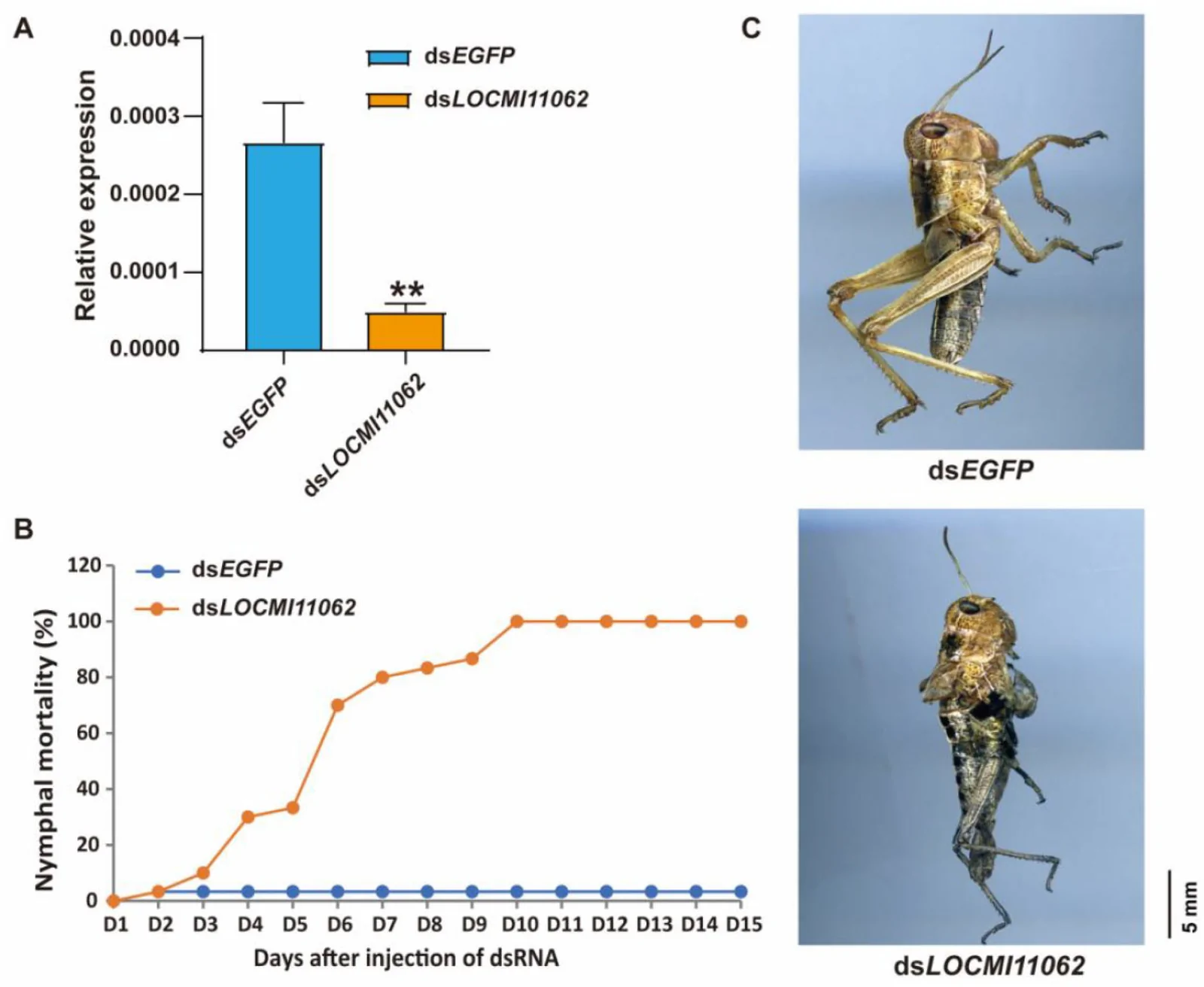
Phenotypic analysis and mortality bioassay after knockdown of *LOCMI11062.* (**A**) Relative expression levels of *LOCMI11062* in integuments after the ds*EGFP* and ds*LOCMI11062* injection were detected by RT-qPCR. *EF1-α* was used as the reference control. (**B**) Mortality of ds*EGFP*- and ds*LOCMI11062*-injected nymphs. All data are reported as means ± SEM of 3 independent biological replications. Statistical significance was analyzed with Mann–Whitney U test. Asterisks indicate significant differences (**, *p* < 0.01). (**C**) Phenotype of the nymphs after ds*EGFP* and ds*LOCMI11062* injection (*n* = 30).

To functionally validate candidate genes identified by transcriptome sequencing following *LmRab11A* knockdown, RNAi was performed for the 9 significantly downregulated DEGs. Among these candidates, silencing of *LOCMI11062* (*β-tubulin*) produced pronounced developmental defects and mortality, whereas knockdown of the remaining genes did not result in obvious phenotypic abnormalities. RT-qPCR confirmed that *LOCMI11062* expression was significantly reduced following ds*LOCMI11062* injection (Figure 3A). Compared with the ds*EGFP* control, locust nymphs injected with ds*LOCMI11062* exhibited progressive mortality, reaching 100% by 10 days after injection (Figure 3B). In addition, all ds*LOCMI11062*-treated nymphs failed to complete molting successfully (Figure 3C).

### 3.4. Effects of LmRab11A on Locust’s Integument Metabolic Chemistry

**Figure 4:**
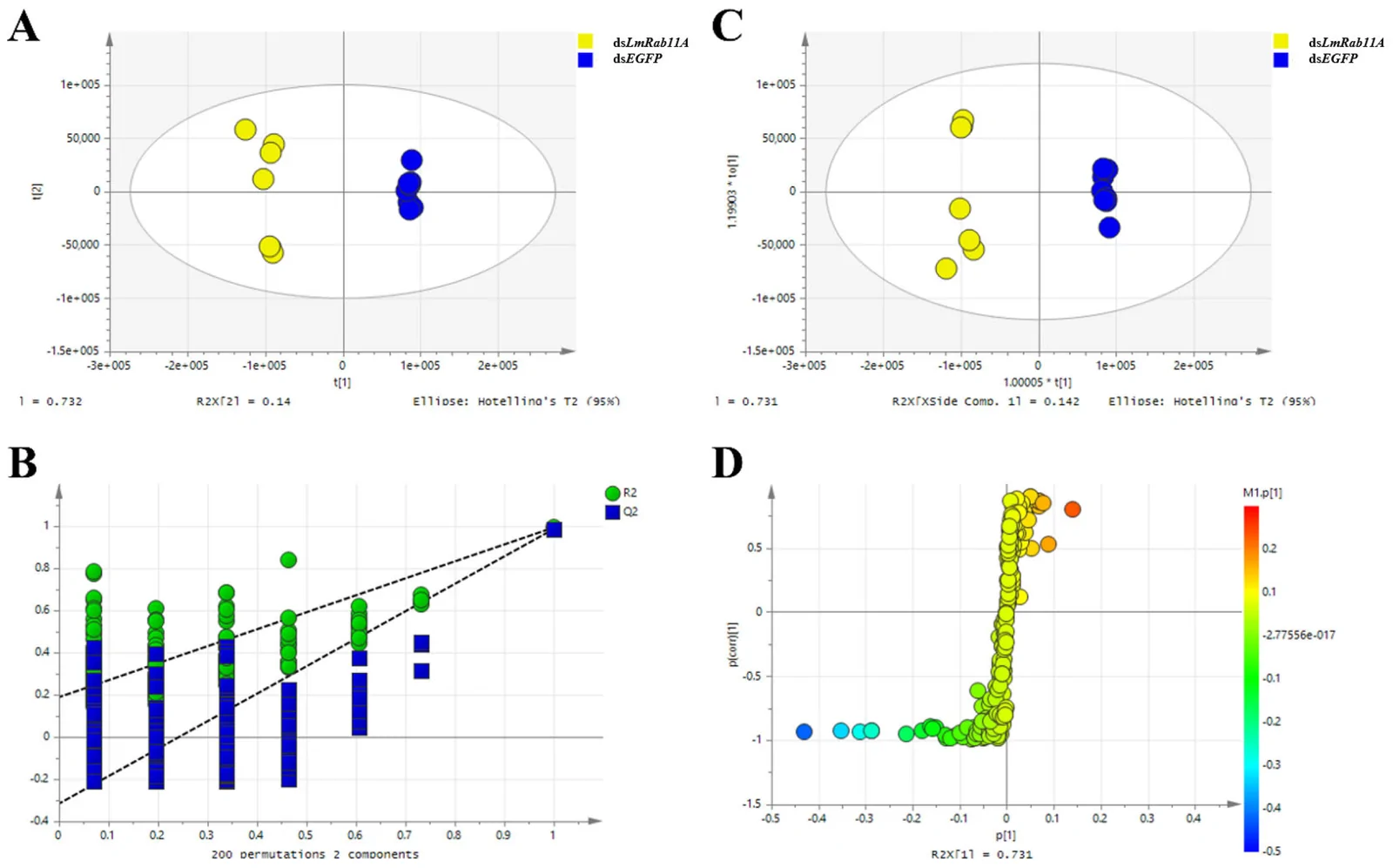
Effects of *LmRab11A* on locust’s integument metabolic chemistry. (**A**) PLS-DA score plot of the different groups of locust’s integument samples. (**B**) The permutation test (permutation no: 200) of locust’s integument samples collected from different groups. The PLS-DA model was validated using the response of the permutation test through 200 permutations, the regression line of the Q2 points intersected the vertical axis (on the left) below zero, and all Q2 values and R2 values to the left were lower than the original points to the right, indicating a low risk of over fitting. (**C**) OPLS-DA score plots of the different groups of locust’s integument samples. (* indicates multiplication) (**D**) S-plots provide visualization of the OPLS-DA-predictive component loading to facilitate model interpretation and reveal the relevant changes in endogenous metabolites responsible for the score plot (*n* = 8). PLS-DA, Partial Least Squares Discriminant Analysis. OPLS-DA, orthogonal projections to latent structures discriminant analysis.

Untargeted metabolomic profiling was performed to investigate the effects of *LmRab11A* knockdown on the metabolic composition of locust integument. Multivariate statistical analysis of the detected metabolites using Partial Least Squares Discriminant Analysis (PLS-DA) revealed a clear separation between the ds*LmRab11A*-treated and control groups, indicating distinct metabolic profiles (Figure 4A). The most notable separation was along the first principal component, suggesting that this primary latent variable effectively distinguishes between the ds*LmRab11A*-treated and control groups. Additionally, both clusters were contained within the ellipse, indicating consistent variations within each group without outliers (Figure 4A). The original model’s Q2 and R2 values were higher than those of the permuted models indicating that the model built on the actual data is significantly better at explaining the variance and predicting the outcomes. The regression line of the Q2 points intersecting the vertical axis below zero suggested that the permuted models were not able to predict well, validating the robustness of the original model (Figure 4B). The OPLS-DA and S-plot loading plot (variable importance in projection value > 1, *p* < 0.05) were used to clarify *LmRab11A*-related differential metabolites (Figure 4C,D).

### 3.5. Effects of LmRab11A on Locust’s Metabolite Profile

**Figure 5:**
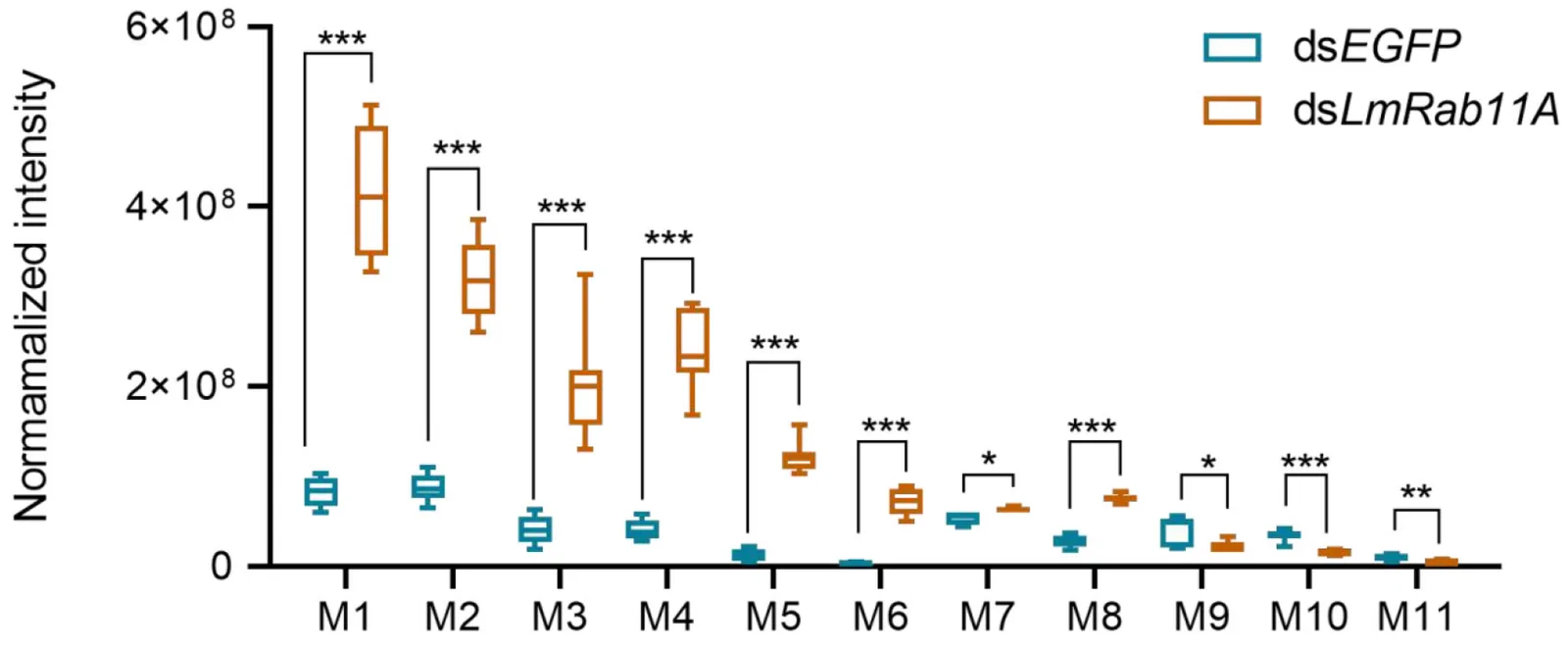
Metabolic profiling changes in locust integument following ds*LmRab11A*-mediated gene silencing. M1: ethyl 1-(4-nitrophenyl)-5-phenyl-3-(2-thienyl)-1H-pyrazole-4-carboxylate; M2: Capryloylglycine; M3: 3-(5-{(3S)-1-[4-(Methylsulfonyl)benzyl]-3-pyrrolidinyl}-1,3,4-oxadiazol-2-yl) pyridine; M4: Sucrose 6-phosphate; M5: (2-morpholino-5-nitrophenyl)methanol; M6: (1R,9S)-11-(2-Furoyl)-5-(2-naphthyl)-7,11-diazatricyclo[7.3.1.02,7]trideca-2,4-dien-6-one; M7: (2E,4E,12Z)-N-Isobutyl-2,4,12-octadecatrienamide; M8: N-Acetyl-L-alanine; M9: NP-011548; M10: NP-002105; M11: Alanyl-Tyrosine. (*, *p* < 0.05; **, *p* < 0.01; ***, *p* < 0.001).

Our findings revealed that RNAi-mediated knockdown of *LmRab11A* affected 11 metabolites. Among these, 8 metabolites exhibited increased abundance following *LmRab11A* silencing, whereas 3 metabolites showed decreased abundance (Figure 5). Of the upregulated metabolites, M2 (Capryloylglycine) and M8 (N-Acetyl-L-alanine) are amino acid conjugates, and M4 (Sucrose 6-phosphate) is a phosphorylated sugar intermediate. The increased levels of these metabolites suggest possible perturbations in amino acid conjugation and carbohydrate metabolism upon *LmRab11A* knockdown. M11 (Alanyl-Tyrosine), a dipeptide, was among the downregulated metabolites, implying that LmRab11A may influence peptide turnover or transport processes. For the remaining differentially accumulated metabolites—namely M1 (ethyl 1-(4-nitrophenyl)-5-phenyl-3-(2-thienyl)-1H-pyrazole-4-carboxylate), M3 (3-(5-{(3S)-1-[4-(Methylsulfonyl)benzyl]-3-pyrrolidinyl}-1,3,4-oxadiazol-2-yl)pyridine), M5 ((2-morpholino-5-nitrophenyl)methanol), M6 ((1R,9S)-11-(2-Furoyl)-5-(2-naphthyl)-7,11-diazatricyclo[7.3.1.02,7]trideca-2,4-dien-6-one), M7 ((2E,4E,12Z)-N-Isobutyl-2,4,12-octadecatrienamide), M9 (NP-011548) and M10 (NP-002105)—their chemical classes and biological functions in insects remain poorly characterized according to current database annotations. Collectively, these results demonstrate that knockdown of *LmRab11A* leads to a broad alteration of the metabolome in *L. migratoria*, affecting metabolites involved in amino acid conjugation, carbohydrate metabolism, and potentially other uncharacterized pathways.

### 3.6. Exploratory Correlation Analysis of Downregulated Genes and Differential Metabolites

**Figure 6:**
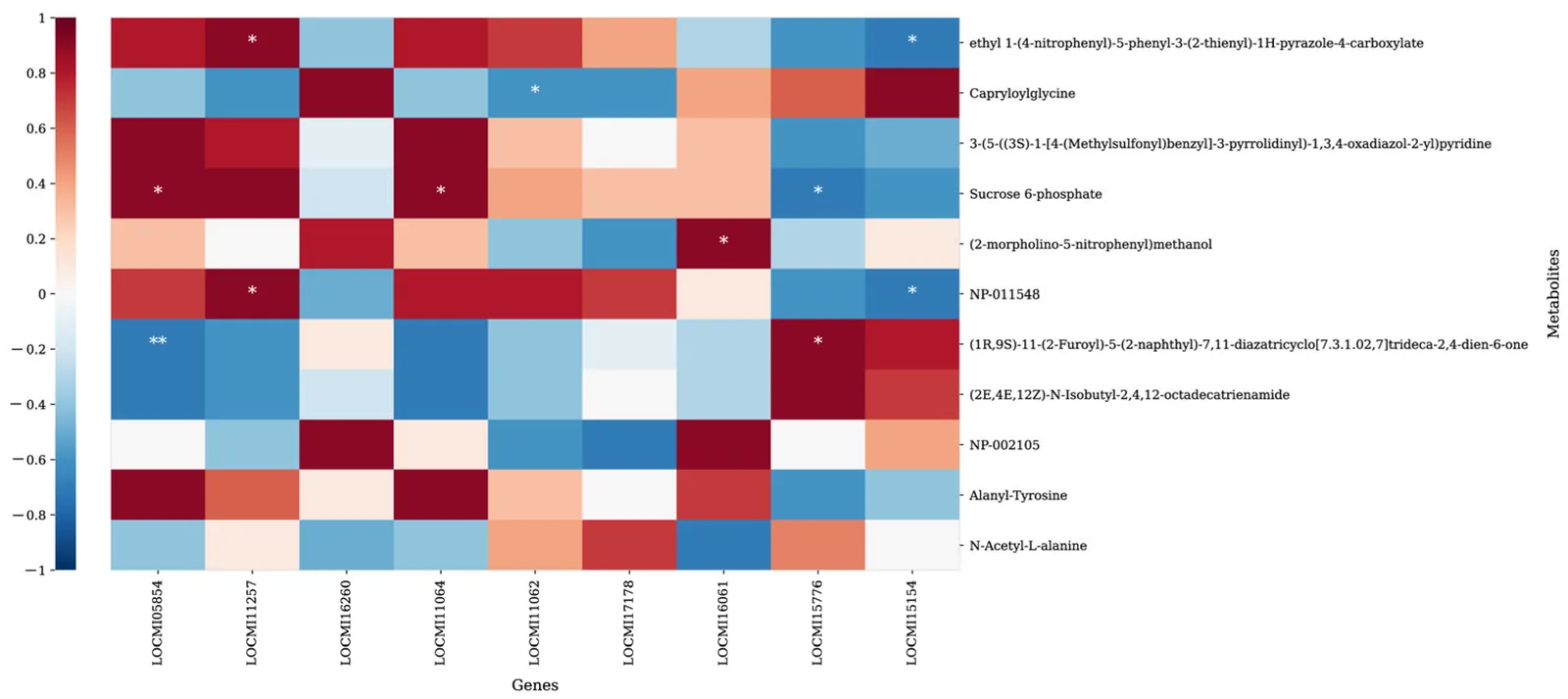
Correlation analysis between the 9 downregulated genes and the 11 altered metabolites.Statistical significance was analyzed with Mann–Whitney U test. Asterisks indicate significant differences (*, *p* < 0.05; **, *p* < 0.01).

To exploratorily screen for potential associations between transcriptomic and metabolomic changes induced by *LmRab11A* knockdown, Spearman’s correlation analysis was performed without multiple testing correction between the 9 significantly downregulated genes and the 11 differential metabolites. As shown in Figure 6, several notable correlations were observed. Specifically, (1R,9S)-11-(2-Furoyl)-5-(2-naphthyl)-7,11-diazatricyclo[7.3.1.02,7]trideca-2,4-dien-6-one was positively correlated with *LOCMI15776* and negatively correlated with *LOCMI5854*. NP-011548 showed a negative correlation with *LOCMI15154* and a positive correlation with *LOCMI11257*. (2-morpholino-5-nitrophenyl)methanol was correlated with *LOCMI16061*. Sucrose 6-phosphate exhibited positive correlations with *LOCMI5854* and *LOCMI11064*, and a negative correlation with *LOCMI15776*. Capryloylglycine was negatively correlated with *LOCMI11062*. Finally, ethyl 1-(4-nitrophenyl)-5-phenyl-3-(2-thienyl)-1H-pyrazole-4-carboxylate demonstrated a positive correlation with *LOCMI11257* and a negative correlation with *LOCMI15154*. These results suggest potential associations between gene expression changes and metabolite abundance following *LmRab11A* knockdown. However, as multiple testing correction was not performed, they do not imply direct regulatory or co-expression relationships.

## 4. Discussion

*Rab11A* has been implicated in the regulation of vesicular trafficking across various species [3]; however, its function in *L. migratoria* remains elusive. In our previous study, injection of ds*LmRab11A* arrested the molting process in locusts. In addition, suppression of *LmRab11A* enhanced RNAi efficiency of the lethal giant larvae (*LmLgl*) [11]. In the present study, we employed transcriptomic and metabolomic approaches to investigate the role of *Rab11A* in *L. migratoria*. We believe that the findings may be applicable across different developmental stages, which will be further explored in future research.

In locust nymphs, the knockdown of the *LmRab11A* gene via RNAi resulted in a robust gene silencing, with a significant 94.9% reduction in *LmRab11A* expression in the integument compared to the control group treated with ds*EGFP*. This high silencing efficiency validates the effectiveness of dsRNA-mediated knockdown in locusts, which is consistent with previous studies that establish RNAi as a powerful tool for gene silencing in insects [11,15,16]. As *Rab* proteins are known to be involved in intracellular vesicle trafficking, the high knockdown efficiency could have profound implications for processes such as membrane recycling and signaling in locusts.

### 4.1. Transcriptomic Analysis of DEGs

The transcriptomic analysis identified 125 DEGs after *LmRab11A* knockdown. Among these, 71 were up-regulated, and 54 were down-regulated. These DEGs were categorized under the following three major gene ontology (GO) categories: cellular components, biological processes, and molecular functions. The most enriched categories were ‘localization’ (biological process), ‘membrane’ (cellular component), and ‘transport activity’ (molecular function), aligning with *Rab11A*’s known role in vesicle trafficking and membrane recycling [17].

Further, COG (Cluster of Orthologous Groups of proteins) analysis highlighted significant roles for carbohydrate and amino acid transport and metabolism and defense mechanisms. Notably, pathway enrichment analysis revealed that DEGs were most significantly involved in glutathione metabolism and ECM–receptor interaction. This observation suggests that *Rab11A* knockdown may impair intracellular trafficking of enzymes crucial to detoxification and oxidative stress management. For instance, reduced glutathione synthesis due to mis-localized enzymes such as glutamate-cysteine ligase (GCL) and glutathione synthetase could elevate oxidative stress levels. This finding is consistent with results from Conde (2014) [18] and Vázquez-Meza (2023) [19], who reported similar disruptions in vesicular transport affecting antioxidant defense mechanisms. Similarly, *Rab11* silencing interferes with ECM–receptor interactions, likely resulting from altered recycling of integrins, which are essential for cell adhesion and communication with the ECM. This may lead to impaired cell signaling, reduced cell migration, and defective ECM remodeling, affecting processes like tissue integrity and immune response [20,21]. These disruptions highlight that *Rab11A* plays a role in maintaining cellular integrity or homeostasis and immune response.

### 4.2. Interplay Between Rab11 and β-Tubulin in Vesicular Trafficking Mechanisms

In this study, we aimed to investigate the phenotypic and biological consequences of RNAi by targeting a subset of down-regulated DEGs in *L. migratoria*. From an initial list of 54 down-regulated DEGs, we selected 15 significantly down-regulated DEGs based on their fold change for further functional validation through RNAi. Among these, only one gene, *LOCMI11062*, demonstrated significant phenotypic effects, including 100% mortality and severe morphological abnormalities in treated locust nymphs. This highlights the critical role of *LOCMI11062* in locust development and survival. Using the NCBI database, we identified *LOCMI11062* as the gene encoding *β*-tubulin, a structural protein fundamental for microtubule formation. Tubulin is a highly conserved protein, and its role in the formation and stabilization of microtubules is well-established across many eukaryotic organisms, including insects [22,23]. Microtubules are involved in essential cellular functions such as mitotic spindle formation, intracellular trafficking, and maintaining cell structure. Knockdown of tubulin genes, therefore, can lead to severe defects in cell division, tissue development, and other physiological processes.

Our findings align with previous studies on tubulin knockdown in various insect species, reinforcing the importance of this protein in insect development. For example, Whyard (2009) [24] reported that γ-tubulin knockdown in *Drosophila melanogaster* led to severe developmental defects in multiple species of *Drosophila*. Similarly, Singh (2013) [25] observed that *β*-tubulin knockdown in *Aedes aegypti* resulted in impaired larval locomotion, reduced growth, and increased mortality, consistent with the effects seen in our study with *L. migratoria*. Another study by Wang (2018) [26] further demonstrated that RNAi targeting α- and *β*-tubulin genes in *Mythimna separata* caused significant mortality, suggesting that tubulin genes are indispensable for insect survival and development.

Despite the functional conservation of tubulin across different species, the effects of its knockdown can vary depending on several factors, such as species specificity, developmental stage, and experimental conditions. For example, while tubulin knockdown in *D. melanogaster* caused developmental defects [24], it did not result in immediate mortality as observed in our study. This variation could be attributed to differences in the role of tubulin in the biology of different species. In *L. migratoria*, injection of dsRNA targeting *β-tubulin* resulted in molting arrest and 100% mortality. By contrast, in the study by [24], delivery of dsRNA targeting tubulin via feeding or soaking led to less severe or delayed lethal effects. While this discrepancy may reflect species-specific differences in the biological role of tubulin, it is important to note that the distinct dsRNA delivery methods employed (injection vs. ingestion/soaking) could also contribute to the observed differences in phenotypic outcomes. Further comparative studies using uniform delivery approaches are needed to determine the relative contributions of species-specific gene function and delivery efficiency to the phenotypic variations.

The link between Rab11 and β-tubulin lies in their roles in intracellular transport. Rab11 regulates vesicular trafficking, particularly recycling endosomes, which depend on the microtubule network formed by *β-tubulin* for movement [27]. Microtubules act as tracks for vesicle transport [28], and disruptions in β-tubulin destabilize this network, impairing vesicular movement. Conversely, Rab11 dysfunction can hinder vesicle attachment and movement along microtubules, leading to impaired trafficking [29,30]. Furthermore, studies have shown that Marburg virus utilizes the Rab11-mediated vesicular transport pathway to release virus-like particles and authentic virions, a process that depends on the microtubule network. Rab11 is closely associated with the microtubule network during its functional activity [31]. Therefore, we hypothesize that knockdown of *Rab11* may induce a corresponding negative feedback response in the microtubule network.

### 4.3. Effects of LmRab11A Knockdown on Integument Metabolism

Metabolomic analysis revealed marked alterations in metabolic profiles following *LmRab11A* knockdown, with 11 key metabolites significantly affected. Among these, eight metabolites showed increased abundance, whereas 3 exhibited decreased abundance. We focused our analysis on the four metabolites (Capryloylglycine, Sucrose 6-phosphate, N-Acetyl-L-alanine, and Alanyl-Tyrosine) that were unequivocally identified. Notably, metabolites such as capryloylglycine and sucrose-6-phosphate displayed significantly correlations with the several down-regulation genes (Figure 5), suggesting that *LmRab11A* silencing impacts metabolic processes, particularly on pathways involved in carbohydrate and amino acid metabolism. Earlier studies have demonstrated that *Rab11* regulates the trafficking and localization of membrane components required for vesicle exocytosis and membrane growth during cellularization, facilitating normal cellular development [32]. We speculate that sucrose-6-phosphate, a critical intermediate in carbohydrate metabolism, may accumulate due to impaired regulation of glycolytic and gluconeogenic pathways resulting from mis-localized enzymes [33]. Similarly, elevated capryloylglycine levels may reflect alterations in lipid metabolism, which are often linked to impaired vesicular transport [34]. In addition, *LmRab11A* knockdown down-regulates certain key metabolites involved in cellular and physiological regulation, including Alanyl-Tyrosine and N-Acetyl-L-alanine. Alanyl-Tyrosine, a di-peptide critical for protein synthesis and neurotransmission, may act as a precursor for tyrosine-based hormones or neurotransmitters essential for initiating hormonal cascades involved in molting [35,36]. Its down-regulation may disrupt these signaling pathways, thereby inhibiting molting and contributing to developmental arrest. N-Acetyl-L-alanine, an acetylated derivative of the non-essential amino acid L-alanine, is involved in amino acid metabolism and may serve as a reservoir for alanine in various physiological processes. Acetylation of amino acids is known to modulate their bioavailability and participate in cellular nitrogen homeostasis and energy metabolism [37]. Although the precise role of N-Acetyl-L-alanine in insect development remains to be fully elucidated, its reduced abundance following *LmRab11A* knockdown suggests a potential disturbance in amino acid utilization and energy balance, which could indirectly impair the metabolic preparation required for successful molting and metamorphosis.

In the present study, Spearman correlation analysis was performed to explore potential associations between the 9 downregulated genes and the 11 differential metabolites following *LmRab11A* knockdown. This analysis was intended solely for exploratory, hypothesis-generating purposes and was not subjected to multiple testing correction. Consequently, the observed correlation should not be interpreted as evidence of co-expression or causal relationships between the identified genes and metabolites. Instead, they represent candidate gene–metabolite associations that provide a framework for generating testable hypotheses. To rigorously evaluate these relationships, future studies should employ targeted functional approaches, including rescue assays, co-RNAi experiments to dissect epistatic interactions among candidate genes, and protein-level validation to determine whether changes at the transcript level are reflected at protein level. In addition, targeted gene manipulation (e.g., knockdown or overexpression of specific candidate genes) combined with quantitative metabolomics will be required to validate the functional links suggested by our correlation matrix. Despite these limitations, our integrated transcriptomic and metabolomic profiling provides a valuable resource for understanding the molecular consequences of *LmRab11A* silencing and offers a foundation for prioritizing candidate gene–metabolite interactions for future functional investigations.

## 5. Conclusions

The RNAi-mediated knockdown of *LmRab11A* in *L. migratoria* has been shown to significantly influence both gene expression and metabolite profiles, disrupting critical metabolic pathways and leading to observable phenotypic changes. These results demonstrate the important role of *LmRab11A* in locust physiology, particularly in cellular metabolism, transport, and molting processes, positioning it as a viable target for pest management strategies. Investigating the identified DEGs and metabolites could further enhance our understanding of the molecular mechanisms underlying locust development and tolerance. The observed correlations between DEGs and metabolites identify essential interactions that may greatly affect locust mortality. However, it is important to note that these correlations are associative rather than causal, and the candidate gene–metabolite links reported here require experimental validation through rescue assays, co-RNAi, and protein-level verification before any causal relationships can be established. Understanding these interactions could provide more insight into the metabolic pathways regulating locust physiology, ultimately leading to the development of targeted RNAi-based pest control strategies.

## Data Availability

The raw transcriptome sequencing data generated in this study have been deposited in the National Center for Biotechnology Information (NCBI) database under submission ID SUB16204729.

## Supplementary Materials

The following supporting information can be downloaded at https://www.mdpi.com/article/10.3390/insects17080803/s1, Figure S1: Relative expression levels of 6 selected DEGs following ds*LmRab11A* injection, as determined by RT-qPCR; Table S1: Primers used in this study for RT-qPCR analysis and dsRNA synthesis.
