# Supplementary material for: Transcriptomic and Metabolomic Analysis Following LmRab11A Knockdown Reveals Its Role in Metabolic Regulation and Molting in Locusta migratoria
Source: Insects. 2026 Aug 3;17(8):803. doi: 10.3390/insects17080803 (PMC13513803; doi:10.3390/insects17080803)
Supplement: Supplementary file 1 [file insects-17-00803-s001.zip › insects-4368107-supplementary.pdf]

Table S1. Primers used in this study for RT-qPCR analysis and dsRNA synthesis.

Primers for RT-qPCR		Size (bp)
LOCMI16061	F: TTTAGTCCCGACTGTATCCTGC	146
	R: GCGATGAACACCCTGAAGC	
LOCMI15776	F: TCGCAATCCTCTGGCACG	143
	R: CAGCAGGTAGTGGTACTGTGGC	
LOCMI15154	F: GCTCTTGCCTTGCGTCTC	157
	R: CGCCTTCAACTGGTCGTG	
LOCMI16260	F: CTACAACTACACCGCCGAGGAC	114
	R: TACGCGATGCCGTTCAGG	
LOCMI17178	F: ACCAGAATGGGCGACGAG	134
	R: GATGTAGAGGATGATGCCGAGA	
LOCMI11257	F: TATCACTTCGGCATATTACC	91
	R: CCACCGCTCTACATTCTC	
LOCMI11062	F:ATCTGAACAGGCTGATTGC	121
	R: TGGATACGAGGGTAGGGA	
LOCMI11064	F: TCCTCAATCACGGCTTCT	178
	R: AGGCGTTTGTTATTTCTGC	
LOCMI05854	F: GGCATTGGACTCGGTTAC	180
	R: ACTGGCATGACTACAAACAAC	
LOCMI16152	F: CGGAGGACATCCAGAAGGTG	84
	R: CGGAGCGGTAGTAGCAGAGC	
LOCMI17246	F: AGCTGAAGGCGGGTGAGA	179
	R: GGTGACGTAAAGCAGGTAGGG	
LOCMI08908	F: GAGTAACACCACAGCCATTCA	149
	R: GAGCAGCAAGGTCTTCACG	
LOCMI11146	F: TTGTCCTCGCCCTTGACG	140
	R: GGTCCCTTGCCATTTCCA	

LOCMI15705	F: AGAAGTACCTGGAGCCCACT	76
	R: ACTGTCACCGAGCACGAGA	
LOCMI16143	F: AATGAAGCCGAAGACAATG	175
	R: CTGGGAGTCCTCAGATGC	
Primers for dsRNA synthesis		
ds*LmEGFP*	F: taatacgactcactataggggacgTAAACGGCCACAAGTT	496
	R: taatacgactcactatagggTGTTCTGCTGGTAGTGGTCG	
ds*LOCMI11062*	F: taatacgactcactatagggGCAGATGCCATCAGACAAGA	486
	R: taatacgactcactatagggTCCAGGGTGGTATGAGTGGT	

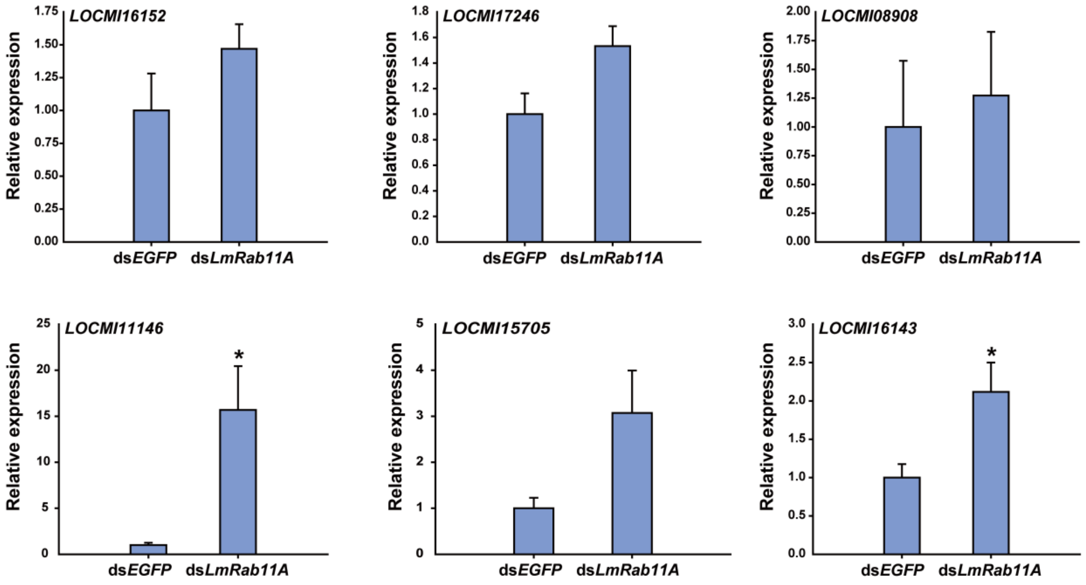

Fig. S1 Relative expression levels of six genes following ds*LmRab11A* injection, as determined by RT-qPCR. Gene expression levels were normalized to the internal control and are presented as mean ± SEM. Statistical analysis was performed using Mann-Whitney U test. Asterisks indicate significant differences compared to the control group (P< 0.05).
